# The Oxidative Stress Product Carboxyethylpyrrole Potentiates TLR2/TLR1 Inflammatory Signaling in Macrophages

**DOI:** 10.1371/journal.pone.0106421

**Published:** 2014-09-03

**Authors:** Ali M. Saeed, Stephanie Duffort, Dmitry Ivanov, Hua Wang, James M. Laird, Robert G. Salomon, Fernando Cruz-Guilloty, Victor L. Perez

**Affiliations:** 1 Sheila and David Fuente Program in Cancer Biology, University of Miami Miller School of Medicine, Miami, Florida, United States of America; 2 Bascom Palmer Eye Institute, Department of Ophthalmology, University of Miami Miller School of Medicine, Miami, Florida, United States of America; 3 Department of Chemistry, Case Western Reserve University, Cleveland, Ohio, United States of America; 4 Department of Microbiology and Immunology, University of Miami Miller School of Medicine, Miami, Florida, United States of America; Massachusetts Eye & Ear Infirmary, Harvard Medical School, United States of America

## Abstract

Oxidative stress is key in the pathogenesis of several diseases including age-related macular degeneration (AMD), atherosclerosis, diabetes, and Alzheimer's disease. It has previously been established that a lipid peroxidation product, carboxyethylpyrrole (CEP), accumulates in the retinas of AMD patients. Retinal infiltrating macrophages also accumulate in the retinas of both AMD patients and in a murine model of AMD. We therefore investigated the ability of CEP-adducts to activate innate immune signaling in murine bone-marrow derived macrophages (BMDMs). We found that CEP specifically synergizes with low-dose TLR2-agonists (but not agonists for other TLRs) to induce production of inflammatory cytokines. Moreover, CEP selectively augments TLR2/TLR1-signaling instead of TLR2/TLR6-signaling. These studies uncover a novel synergistic inflammatory relationship between an endogenously produced oxidation molecule and a pathogen-derived product, which may have implications in the AMD disease process and other oxidative stress-driven pathologies.

## Introduction

Oxidative damage is a key pathological finding in states of disease and the general aging process. The molecular targets of oxidative damage include nucleic acids, proteins, and lipids. These oxidatively modified molecules can serve as markers of oxidative stress or they may themselves aid in further tissue destruction, promoting a pathological inflammatory response. Lipid peroxidation products, in particular, have been identified to function in such a manner and are capable of activating pattern recognition receptors (PRRs) on innate immune cells [1]. In this respect, certain oxidized lipids function as damage associated molecular patterns (DAMPs) and have been termed “oxidation-specific epitopes” [2]–[4]. PRRs on innate immune cells detect danger signals, whether they are from invading pathogens (via pathogen associated molecular patterns [PAMPs]) or endogenously derived products (DAMPs). DAMPs can be intracellular proteins that leak into the extracellular space (e.g., Hsp70, HMGB1) or molecules that become altered to form neo-epitopes. As an example, the prototypical PRR toll-like receptor 4 (TLR4) expressed by macrophages can bind bacterially-derived lipopolysaccharide (LPS) or endogenously generated minimally oxidized-low density lipoprotein (mmLDL), which leads to macrophage activation and release of inflammatory cytokines [5], [6]. Other oxidized lipid products implicated as DAMPs include malondialdehyde (MDA), oxidized 1-palmitoyl-2-arachidonoyl-sn-3-glycero-phosphorylcholine (oxPAPC), and carboxyethylpyrrole (CEP) [1], [7]–[9]. These oxidized lipids have been shown to induce inflammatory cytokine production in macrophages.

Our previous work has focused on CEP, which is formed from its precursor docosahexanoic acid under conditions of oxidative stress. CEP condenses with certain amino acid residues forming covalent oxidized lipid-protein adducts [10]. Elevated levels of CEP-adducts are found in several pathological states including cancer, autistic brain tissue, atherosclerotic plaques, sites undergoing wound healing, and in AMD patients’ retinas [10]–[15]. Using a mouse model of AMD that utilizes CEP-immunization, we previously reported that in addition to signs of retinal damage mimicking AMD pathology in humans, CEP-immunized mice develop retinal infiltrating macrophages [16], [17]. In characterizing these macrophages we distinguished their polarization to be M1, not M2. Human AMD donor eyes also contain retinal M1-skewed macrophages as compared to aged non-AMD subject retinas, which had M2 macrophage accumulation [18]. M1 macrophages are pro-inflammatory (producing TNF-α, IL-12) and can induce tissue damage, while M2 macrophages are anti-inflammatory (producing IL-10) and can induce tissue repair [19]. In addition to AMD, the coincident accumulation of CEP-adducts and macrophages occurs in atherosclerotic plaques (foam cells), cancer (tumor-associated macrophages), and wound healing (M2-macrophages). Thus, understanding the CEP/macrophage relationship could shed light on the pathogenesis of several highly-prevalent diseases.

In this work we investigated the innate immune response of macrophages to CEP, asking if CEP promotes inflammatory signaling. The rationale for this inquiry was based on the previous discovery that CEP activates the innate receptor TLR2 to induce endothelial angiogenesis [15]. More recently, CEP was shown to induce inflammasome priming (via TLR2) in macrophages, promoting IL-1β release [7]. However, CEP was shown ineffective for promoting TNF-α secretion, a non-inflammasome dependent cytokine [7]. In the present study, we sought to determine if CEP could possibly activate TNF-α and we were particularly interested in how CEP-adducts might modulate the various inflammatory mediators (e.g., cytokines, DAMPs, PAMPs) found in the extracellular milieu at sites of inflammation. Our data demonstrate for the first time that CEP-adducts cooperate in a highly specific manner to amplify low-grade inflammation mediated by TLR2/TLR1-activating PAMPs and augments M1 polarization. These findings have broad implications for oxidative stress-driven pathologies, where CEP may exacerbate deleterious inflammatory signaling.

## Materials and Methods

### Materials

CEP-adduction of proteins was prepared from commercially available highly purified cell-culture grade bovine serum albumin, human serum albumin, and transferrin (Sigma-Aldrich, St. Louis, MO). Proteins were CEP-adducted following previously published procedures [20]. Chromogenic limulus amebocyte lysate assay (Lonza, Walkersville, MD) was conducted to ensure protein preparations were endotoxin free (i.e., had completely undetectable endotoxin levels). Proper CEP adduction was confirmed using ELISA and western blot. All of the TLR-agonists, heat-killed bacteria, ATP, anti-TLR1 (rat polyclonal) antibody, and PMA were purchased from Invivogen (San Diego, CA). IL-4 was purchased from eBiosciences (San Diego, CA). MDA-BSA was kindly provided by Cristoph Binder (Medical University of Vienna). Hsp70 was purchased from Enzo Life Sciences (Farmingdale, NY) and HMGB1 was purchased from HMGBiotech (Milano, Itlay). The anti-CEP antibody used for the immuno-depletion experiments was produced by our lab in conjunction with Genscript (Piscataway, NJ) and was tested for specificity against a variety of CEP-modified proteins and lacked recognition to albumin and several other lipid-derived modifications.

### Mice

BALB/c and C57BL/6 wild-type mice were obtained from The Jackson Laboratory. *Tlr4−/−* (C57BL/6) mice were kindly provided by Dr. Dmitry Ivanov (Bascom Palmer Eye Institute). Protocols for use of experimental animals in this study adhered to the ARVO Statement for the Use of Animals in Ophthalmic and Vision Research and were approved by the Institutional Animal Care and Use Committee of the University of Miami Miller School of Medicine.

### Bone-marrow derived macrophages (BMDMs) and cell culture

All experiments were performed using BMDMs from BALB/c mice, except the *Tlr4−/−* derived BMDMs which were in the C57BL/6 background. For this experiment Tlr4−/− derived BMDMs were compared with BMDMs from wild-type C57BL/6. Comparison between wild-type BALB/c and wild-type C57BL/6 derived BMDMs showed equivalent behavior (Fig. S1). Bone marrow aspirated cells from mice were used to generate BMDMs. Aspirated cells were plated at density of 1 million per well of a six-well plate. Cells were differentiated in DMEM (Life Technologies, Carlsbad, CA) supplemented with 10% heat-inactivated FBS (Atlanta Biologicals, Atlanta, GA) and 15% L929 (ATCC, Rockville, MD) cell-conditioned media (as a source of M-CSF) for seven days. Macrophage culture purity was determined by flow cytometry and cultures were >97% double positive for F4/80 and CD11b. Media was replaced on day seven and BMDMs were stimulated in fresh DMEM as indicated in the figure legends (CEP-BSA, sham-BSA concentration was 10 µg/mL if not specified). THP-1 (ATCC TIB-202) cells were differentiated into macrophages with PMA (50 ng/mL) incubation for 3 days.

### Real-time quantitative PCR (qPCR)

Total RNA was isolated from cells using the RNeasy kit (Qiagen, Valencia, CA), then cDNA was generated with the Maxima First Strand cDNA Synthesis Kit (Thermo Scientific, Waltham, MA). Gene expression was measured by real-time quantitative PCR (qPCR) using the indicated primers. qPCR was performed using SYBR Green Supermix (Bio-Rad, Hercules, CA) on a Roche Light Cycler real-time PCR instrument (Indianapolis, IN). Relative gene expression was calculated using the ΔΔC_t_ method, with gene expression normalized to *Gapdh* expression. Each treatment is represented as relative-expression (i.e., fold-expression over reference group), where the control sample served as the reference with a set value of 1. A two-tailed Student’s t test was used for statistical analysis. The following primers were used (forward primer listed first and reverse primer listed second): Gapdh (AGGTCGGTGTGAACGGATTTG; TGTAGACCATGTAGTTGAGGTCA), Tnf (CTGAACTTCGGGGTGATCGG; GGCTTGTCACTCGAATTTTGAGA), Il12a (CAATCACGCTACCTCCTCTTTT; CAGCAGTGCAGGAATAATGTTTC), Arg1 (CTCCAAGCCAAAGTCCTTAGAG; AGGAGCTGTCATTAGGGACATC), Il10 (GCTCTTACTGACTGGCATGAG; CGCAGCTCTAGGAGCATGTG), Hmox1 (AAGCCGAGAATGCTGAGTTCA; GCCGTGTAGATATGGTACAAGGA), Srxn1 (CCCAGGGTGGCGACTACTA; GTGGACCTCACGAGCTTGG), Nos2 (GTTCTCAGCCCAACAATACAAGA; GTGGACGGGTCGATGTCAC), Il6 (TAGTCCTTCCTACCCCAATTTCC; TTGGTCCTTAGCCACTCCTTC), hTNF (CCTCTCTCTAATCAGCCCTCTG; GAGGACCTGGGAGTAGATGAG), hGAPDH (GGAGCGAGATCCCTCCAAAAT; GGCTGTTGTCATACTTCTCATGG).

### ELISA

TNF-α, IL-12, and IL-1β protein levels in BMDM cultures supernatants were quantified by sandwich ELISA. Appropriate antibody pairs and recombinant TNF-α and IL-12 protein (for standards) were all purchased from eBiosciences (San Diego, CA). Binding of biotinylated secondary antibodies were quantified using strepavidin-alkaline phosphatase with phophatase substrate solution (Sigma-Aldrich, St. Louis, MO). Absorbance was read on a plate reader and cytokine concentrations were extrapolated based on generating a standard curve with the recombinant proteins. A two-tailed Student’s t test was used for statistical analysis.

### Immuno-depletion of CEP-BSA and TLR1 neutralization

A 200 uL PBS solution containing 1 µg CEP-BSA (or sham-BSA), anti-CEP antibody, and protein A/G agarose beads (Santa Cruz Biotechnology, Santa Cruz, CA) was incubated overnight on an inverting rocker. Then, the beads were pelleted by brief centrifugation and the supernatant was used for BMDM-stimulation. Control pull-downs with IgG1-isotype control antibody were also performed. For TLR1 antibody neutralization experiments, THP-1 cells were pre-incubated with anti-TLR1 antibody or isotype control (10 µg/mL final) for 3 hours.

### Online Supplemental Material

Online supplemental figures include Figure S1, which demonstrates a comparison between wild-type BALB/c and wild-type C57BL/6 BMDMs. Figure S2 shows sequential CEP and Pam stimulation as well as wash out experiments. Figure S3 depicts how various concentrations of LPS-E. Coli induce macrophage TNF-α gene expression. Figure S4 demonstrates CEP-BSA co-stimulation with various concentrations of LPS-E. Coli.

## Results

### CEP potentiates low-dose Pam3CSK4-induced inflammatory cytokine production

**Figure 1:**
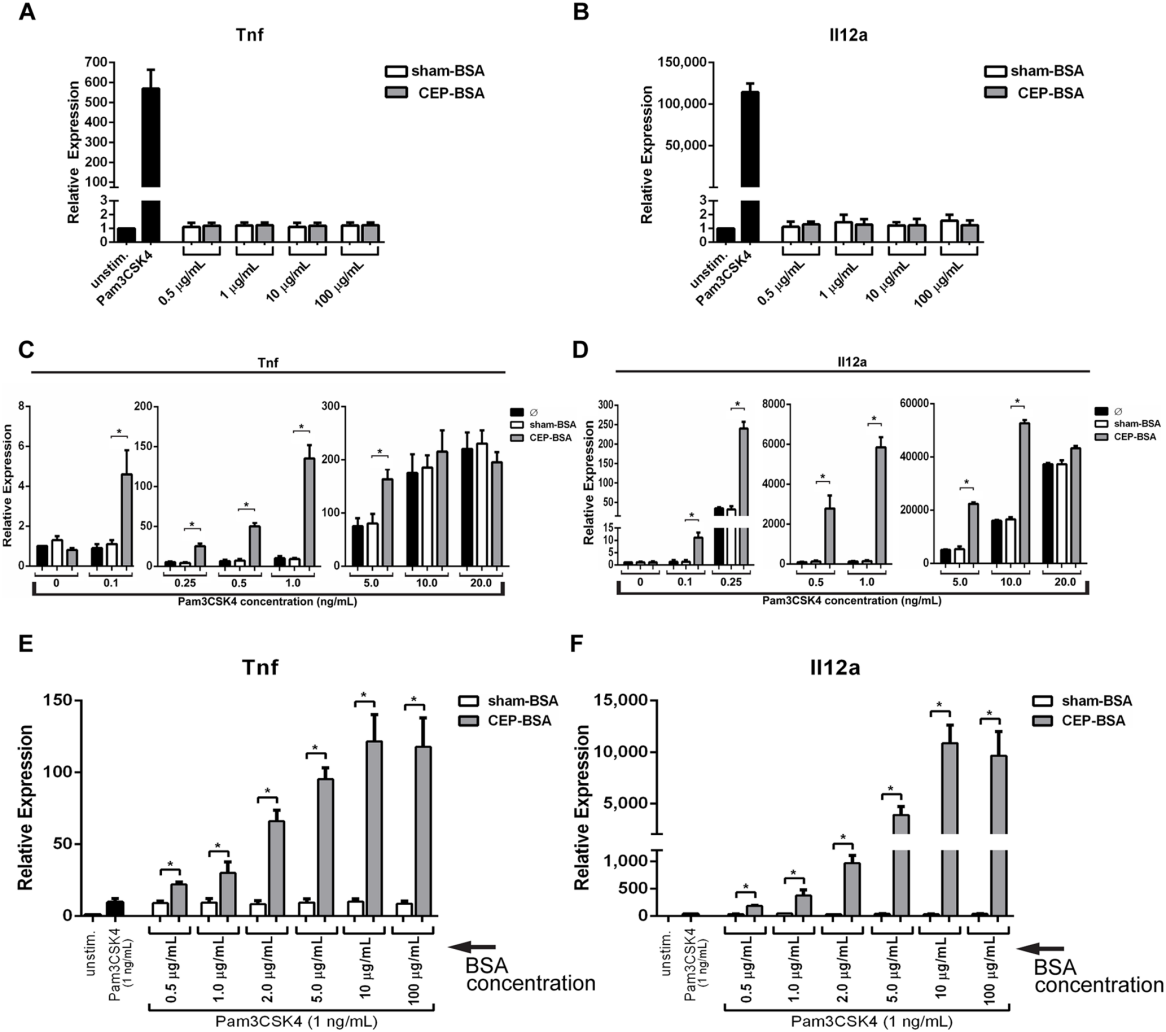
CEP synergizes with the TLR2-agonist Pam3CSK4 to induce inflammatory gene expression. ***(A, B, C, D, E, F)*** BMDMs were stimulated with the indicated treatment. After stimulation relative expression of *Tnf* or *Il12a* (as indicated) was determined by qPCR. (**A, B**) BMDMs were stimulated for 9 hours with Pam3CSK4 (500 ng/mL), the indicated concentration of sham-BSA or CEP-BSA, or left unstimulated (n = 5). (**C, D**) BMDMs were stimulated with various concentration of Pam3CSK4 from 0.1–20 ng/mL (as indicated) and were co-stimulated with either nothing (Ø), sham-BSA (100 µg/mL), or CEP-BSA (100 µg/mL) for 9 hours (n = 6). (**E, F**) BMDMs were stimulated with Pam3CSK4 (1.0 ng/mL) and varying concentrations of sham-BSA or CEP-BSA (as indicated) (n = 3). *P<0.05. Results are mean +/− SEM.

In a CEP-immunized mouse model of AMD, we previously found retinal infiltrating macrophages that expressed TNF-α and IL-12 [16]. Given that the retina is highly abundant in CEP, in a recent report we sought to determine if CEP can directly induce expression of these two inflammatory cytokines in macrophages [11], [13], [21], [22]. In that report, we found that macrophages stimulated with CEP-adducted albumin secreted TNF-α and IL-12. Although we had documented a CEP-specific response (relative to albumin control), we always noted a background level of stimulation by the albumin alone (relative to unstimulated control). We therefore obtained highly purified albumin that was subjected to extra purification steps (e.g., salt fractionation, ion exchange, and gel filtration chromatography) in an effort to eliminate the background level of stimulation by albumin alone. This higher purity albumin was CEP-adducted and we then stimulated murine bone-marrow derived macrophages (BMDMs) with various concentrations of either CEP-adducted bovine serum albumin (CEP-BSA) or sham-BSA. Sham-BSA represents BSA that was mock adducted. The TLR2 agonist Pam3CKS4 served as a positive control since CEP has been reported to signal via TLR2 [7], [15]. Inflammatory gene expression was assessed by qPCR analysis of *Tnf* and *Il12a* mRNA levels. As demonstrated in Figure 1A and 1B, in contrast to our positive controls, concentrations of up to even 100 µg/mL of CEP-BSA failed to elicit gene induction of *Tnf* or *Il12a*. Importantly absent was the background level of stimulation by albumin alone.

Given that CEP has been reported to induce TLR2-dependent cell-signaling in macrophages and endothelial cells, we speculated that although CEP may not directly induce inflammatory gene transcription on its own, it may instead cooperate with TLR2-mediated inflammatory signaling [7], [15]. To test this hypothesis, we co-stimulated BMDMs with varying concentrations of Pam3CSK4 and either sham-BSA or CEP-BSA and measured inflammatory cytokine gene expression. The data from these experiments demonstrate for the first time that at sub-maximal concentrations (0.1–5.0 ng/mL) of Pam3CSK4, CEP strongly potentiates the Pam3CSK4-induced gene expression of *Tnf* and *Il12a* (Fig. 1C and 1D). Moreover, at concentrations where Pam3CSK4 (i.e., 0.1 ng/mL) did not elicit observable gene expression, CEP was still able to potentiate mRNA expression of *Tnf* and *Il12a*. Optimal synergy was observed at a Pam3CSK4 concentration of 1 ng/mL. At this concentration, CEP offered an approximately 13-fold enhancement for *Tnf* mRNA expression and an approximately 40-fold enhancement for *Il12a* expression when compared with Pam3CSK4 alone. To determine the optimal CEP concentration, we next co-stimulated BMDM with a fixed concentration of Pam3CSK4 (at 1.0 ng/mL) and varying concentrations of CEP-BSA or sham-BSA (from 0.5 µg/mL to 100 µg/mL) and again measured *Tnf* and *Il12a* mRNA expression. The synergy between Pam3CSK4 and CEP was observed at concentrations as low as 0.5 µg/mL of CEP-BSA (Fig. 1E and 1F). Importantly, CEP induced synergy with Pam3CSK4 in a dose-dependent manner, with maximal gene induction occurring at 10 µg/mL of CEP-BSA. In addition, sequential stimulation with CEP pre-incubation leads to stronger synergy than co-stimulation, suggesting that CEP-specific signaling is a rather active process (Fig. S2).

**Figure 2:**
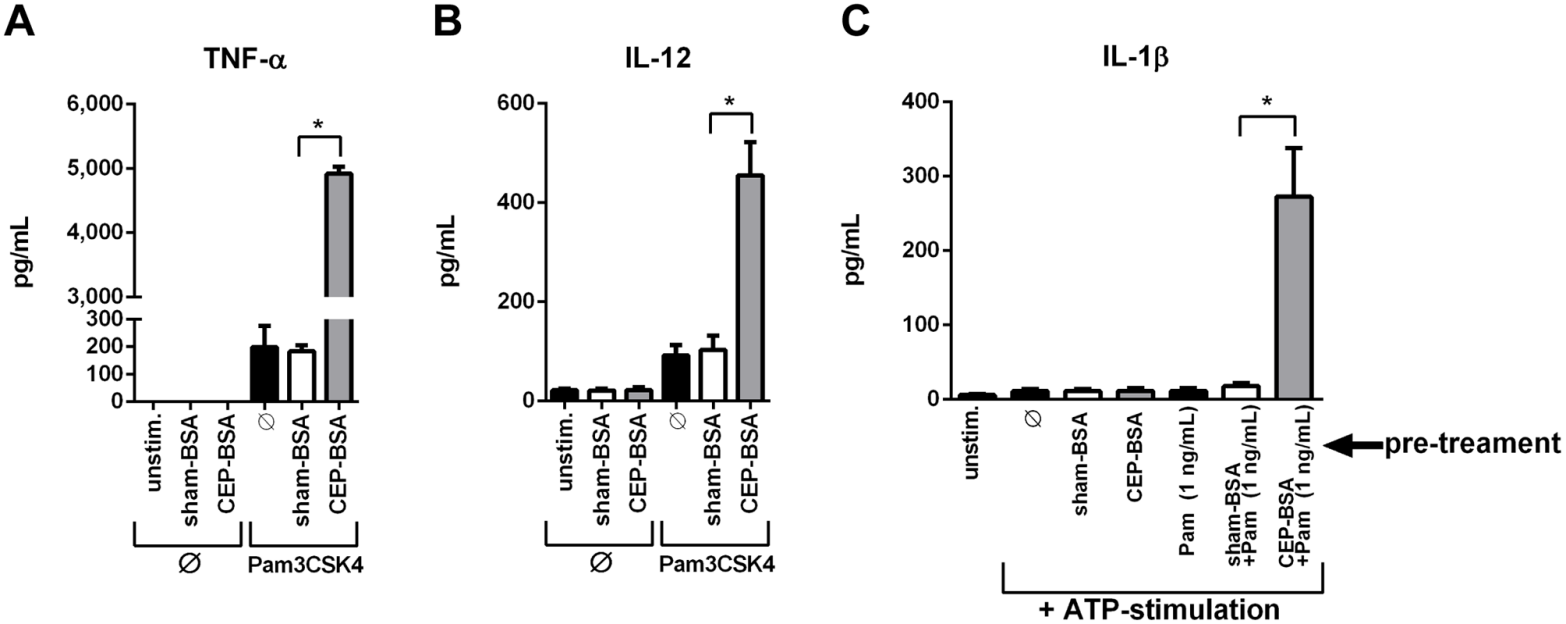
CEP and Pam3CSK4 synergize to induce secretion of inflammatory cytokines. (**A, B**) ELISA was performed for TNF-α and IL-12. BMDMs were co-stimulated for 24 hours as indicated (Pam3CSK4 [1.0 ng/mL], sham-BSA [10 µg/mL] or CEP-BSA [10 µg/mL]) (n = 3). (**C**) ELISA for IL-1β. BMDMs were pretreated (as indicated) with sham-BSA, (10 µg/mL), CEP-BSA (10 µg/mL), and/or Pam3CSK4 (1.0 ng/mL) for 20 hours. After this, cells were stimulated with ATP (5 mM) for 6 hours. (n = 3) *P<0.05. Results are mean +/− SEM.

To corroborate the qPCR mRNA expression analysis with protein production, ELISA analysis of TNF-α and IL-12 was performed. Similar to *Tnf* and *Il12a* mRNA induction, CEP alone was not able to induce secretion of either TNF-α or IL-12 (Fig. 2A and 2B). However, mirroring the qPCR data, the combination of CEP-BSA and Pam3CSK4 induced a robust production of both TNF-α and IL-12, to levels far greater than Pam3CSK4 alone (Fig. 2A and 2B). We also determined the ability of CEP-BSA to induce secretion of IL-1β, an important inflammatory cytokine that relies on the inflammasome, which is a multimeric protein complex with protease activity. Inflammasome-mediated IL-1β secretion can be experimentally induced by pre-treating macrophages with an inflammasome priming agent (such as LPS) followed by stimulation with ATP [23]. Recently Doyle et al. demonstrated that CEP-HSA (human serum albumin) can trigger inflammasome activation, acting as a priming stimulus that promotes the ATP-dependent release of IL-1β [7]. As shown in Figure 2C, priming with CEP-BSA alone (followed by ATP-stimulation) failed to elicit IL-1β secretion. However, priming with CEP-BSA and low-dose Pam3CSK4 together (followed by ATP-stimulation) lead to synergistic IL-1β production (Fig. 2C).

Taken together these results demonstrate an entirely novel synergistic inflammatory relationship between an endogenously produced oxidation product and a bacterial PAMP.

### CEP is a unique oxidized lipid-protein adduct and selectively enhances Pam3CSK4-signaling

**Figure 3:**
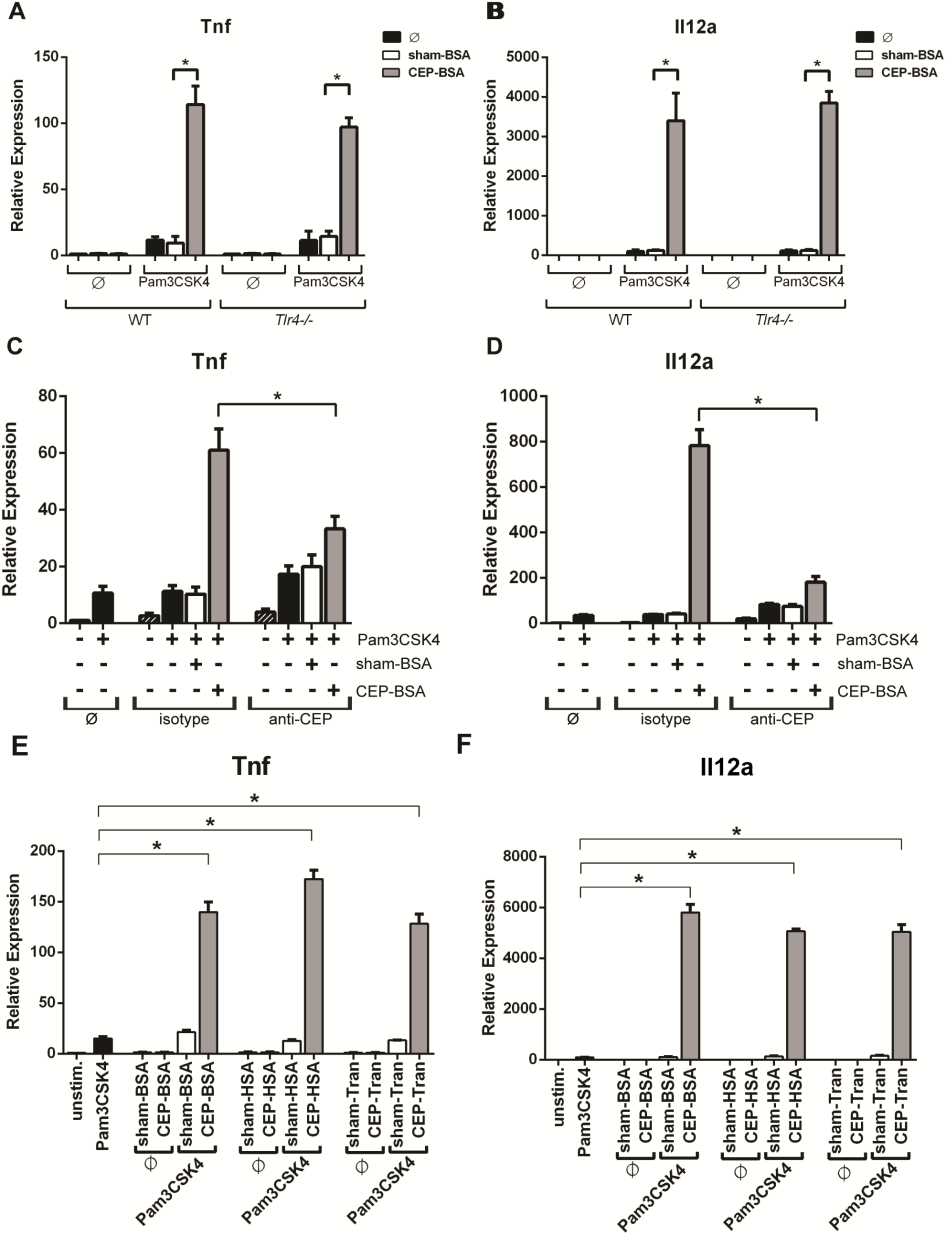
CEP selectively enhances Pam3CSK4-signaling in a TLR4-independent manner. (**A, B**) BMDMs from either wild-type (WT) or *Tlr4−/−* mice were co-stimulated as indicated with Pam3CSK4 (1.0 ng/mL) and sham-BSA (10 µg/mL) or CEP-BSA (10 µg/mL) for 9 hours. *Tnf* or *Il12a* expression was assessed by qPCR (n = 3). (**C, D**) Immuno-depletion assays were performed as described in the Methods & Materials section. sham-BSA (1 µg/mL) or CEP-BSA (1 µg/mL) were immuno-depleted with either IgG1-isotype control or anti-CEP antibody. Supernatants were either combined or not with Pam3CSK4 (1.0 ng/mL) for stimulation of BMDMs. *Tnf* or *Il12a* expression was assessed by qPCR (n = 3). (**E, F**) BMDMs were stimulated with the indicated combinations of Pam3CSK4 (1 ng/mL) and either sham-adducted (10 µg/mL) or CEP-adducted (10 µg/mL) BSA, HSA, or transferrin (Tran) for 9 hours followed by qPCR expression analysis of *Tnf* and *Il12* (n = 3) *P<0.05. Results are mean +/− SEM.

Since we are studying innate immune signaling, it is important to rule out contaminants in our CEP preparations, especially endotoxin (i.e., LPS) contamination. Figure 1 already suggests the high purity of our CEP preparations because even 100 µg/mL of CEP-BSA failed to elicit inflammatory gene activation (Fig. 1A and 1B). To further exclude the possibility of endotoxin contamination, we tested the synergy between CEP and Pam3CSK4 using BMDMs derived from *Tlr4−/−* mice (because TLR4 is the receptor for endotoxins). *Tlr4−/−* BMDMs show that CEP robustly enhances Pam3CSK4-induced *Tnf* and *Il12a* expression, to the same extent observed in wild-type BMDMs (Fig. 3A and 3B). This indicates the enhancing activity provided to Pam3CSK4 by CEP is not due to endotoxin contamination. As an additional measure to rule out contaminants, we immuno-depleted CEP-adducts from our CEP-BSA preparations using a CEP-specific antibody. After immuno-depletion of the CEP-BSA preparation, we stimulated cells with Pam3CSK4 and the immuno-depleted solution of CEP-BSA. The data from these experiments demonstrate that immuno-depletion with anti-CEP antibody (but not isotype antibody) strongly attenuates the ability to potentiate Pam3CSK4-induced *Tnf* and *Il12a* gene expression (Fig. 3C and 3D). We next wanted to confirm that the observed CEP potentiation was independent of the carrier protein and performed experiments using CEP adducted to human serum albumin (HSA) and transferrin (Tran). Both CEP-HSA and CEP-Tran robustly augmented low-dose Pam3CSK4 signaling to a similar extent as CEP-BSA (Fig. 3E and 3F). This result demonstrates that CEP (and not the protein carrier) is truly responsible for the observed synergistic behavior. This result, along with the results from the *Tlr4*−/− BMDMs and the immuno-depletion experiments help rule out contaminants and activity from the protein carrier as being responsible for our observed CEP-signaling.

**Figure 4:**
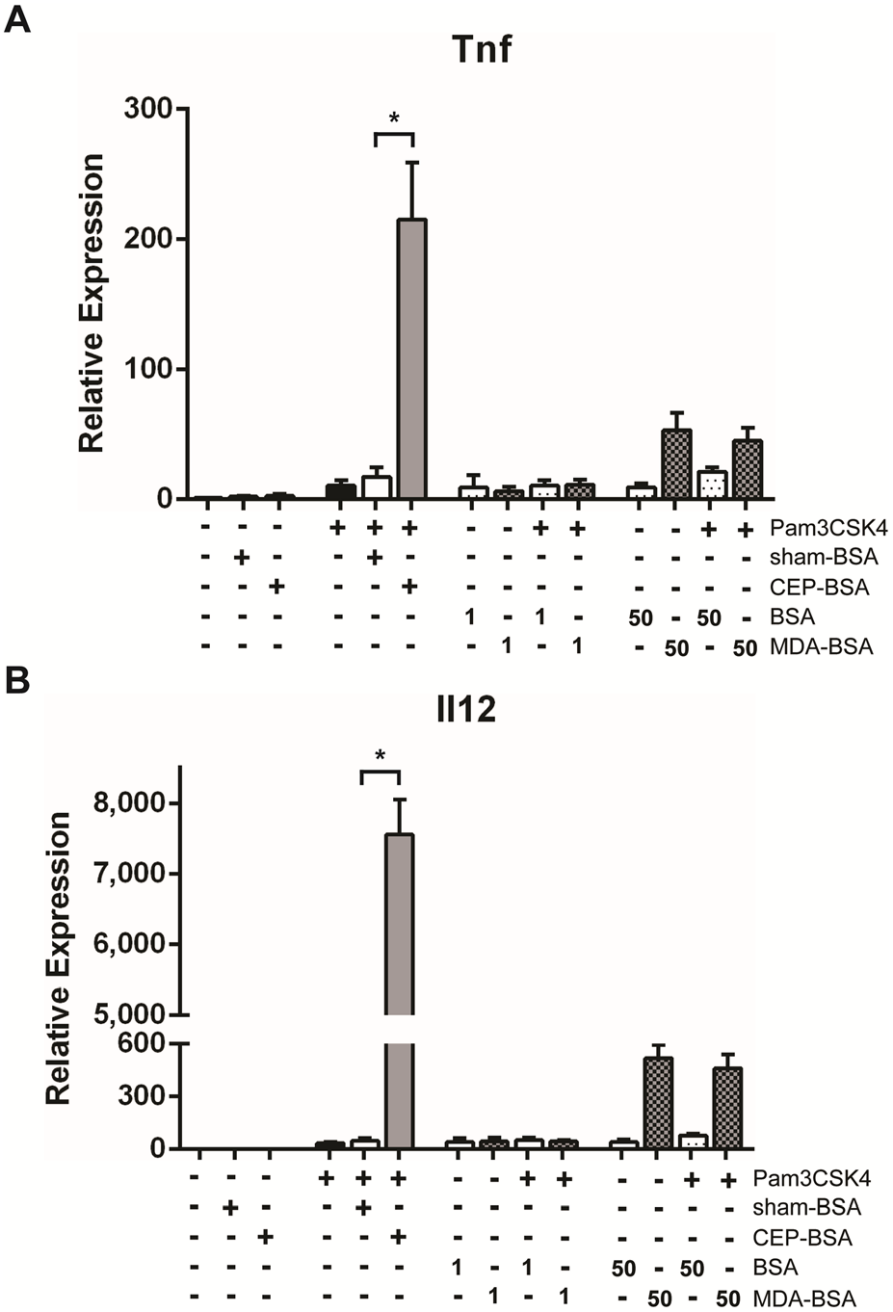
Malondialdehyde (MDA), another lipid peroxidation product, does not potentiate Pam3CSK4. (**A, B**) BMDMs were stimulated for 9 hours with indicated combination(s) of Pam3CSK4 (1.0 ng/mL), sham-BSA (10 µg/mL), CEP-BSA (10 µg/mL), BSA (at the indicated concentration in µg/mL) and/or MDA-BSA (at the indicated concentration in µg/mL). *Tnf* or *Il12a* expression was assessed by qPCR (n = 3). *P<0.05. Results are mean +/− SEM.

We next tested if an oxidized lipid other than CEP can augment Pam3CSK4 signaling in order to determine if CEP’s potentiation of Pam3CSK4-singaling was a general feature of oxidized lipid adducts or a unique property of CEP. We tested the ability of malondialdehyde-adducted BSA (MDA-BSA) to synergize with Pam3CSK4. MDA has been implicated in the pathogenesis of both AMD and atherosclerosis [9]. BMDMs were co-stimulated with 1.0 ng/mL of Pam3CSK4 and either BSA or MDA-BSA (at 1 or 50 µg/mL). Figure 4A and 4B demonstrate that MDA-BSA fails to enhance Pam3CSK4-mediated *Tnf* and *Il12a* gene expression. These results demonstrate that Pam3CSK4’s potentiation by CEP exhibits specificity and that the observed CEP/Pam3CSK4 synergy cannot be elicited by just any other oxidized-lipid protein adduct.

### CEP potentiates an M1 and not an M2 or Mox macrophage phenotype

**Figure 5:**
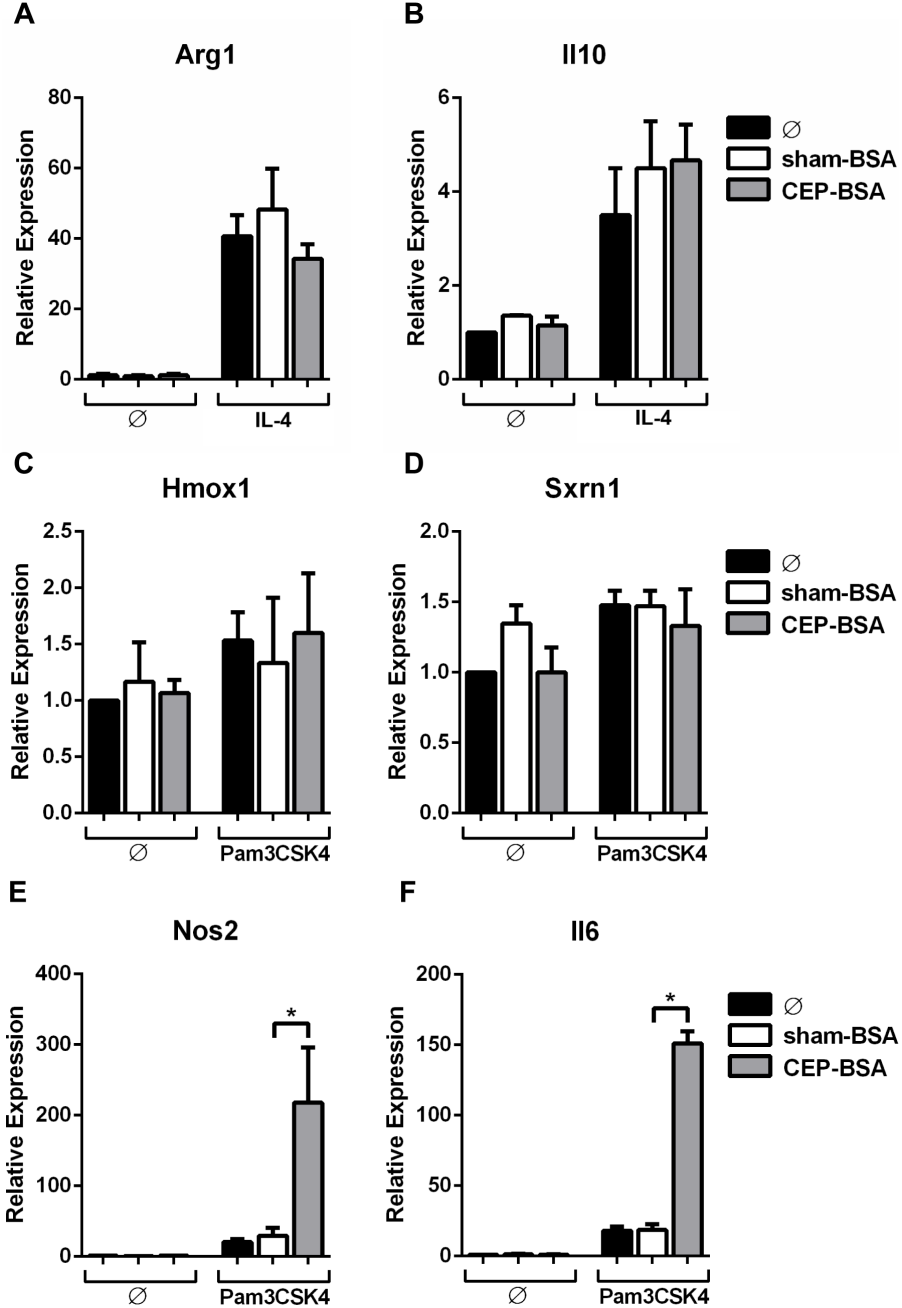
CEP promotes M1 polarization and does not aid in M2 or Mox polarization. (**A, B**) BMDMs were stimulated as indicated with either sham-BSA (10 µg/mL), CEP-BSA (10 µg/mL), and/or IL-4 (1.5 ng/mL) for 9 hours. (**C, D, E, F**) BMDMs were stimulated as indicated, sham-BSA (10 µg/mL), CEP-BSA (10 µg/mL), and Pam3CSK4 (1.0 ng/mL) for 9 hours (n = 6). For (**A, B, C, D, E and F**) The indicated mRNA was assessed by qPCR. *P<0.05. Results are mean +/− SEM.

CEP synergizes with Pam3CSK4 to induce expression of *Tnf* and *Il12a* mRNA, which are markers of M1-macrophages. It is worth noting that Pam3CSK4 will by itself promote M1 polarization. It is possible that CEP is merely pushing a macrophage towards a subset to which it is already slightly skewed. Therefore, we sought to determine if CEP can promote M2 polarization if combined with a low-grade M2-inducing stimulus. As mentioned, M2 macrophages accumulate in the aging retina of non-AMD subjects [18]. IL-4 is a known M2-inducer characterized by promoting the expression of *Il10* and *Arg1* transcripts [19]. Therefore, we co-stimulated BMDMs with low-dose IL-4 and CEP-BSA. We did not observe potentiation of either *Arg1* or *Il10* mRNA induction with CEP-BSA and IL-4 co-stimulation (Fig. 5A and 5B). Also, CEP did not induce M2-polarization by itself. Next we examined the ability of CEP to induce a newly described macrophage polarization state termed Mox [24]. Mox macrophages develop in response to stimulation by oxidized lipids and are characterized by the upregulation of a unique set of transcripts (e.g., *Hmox1, Srxn1*), which are not found in M1 or M2 macrophages [24]. We found that macrophages stimulated with CEP-BSA did not upregulate Mox-genes such as *Hmox1* and *Srxn1* (Fig. 5C and 5D). Additionally, we did not observe induction of these genes with the co-stimulation of CEP-BSA and Pam3CSK4. Lastly, to determine if Pam3CSK4 and CEP synergize to promote the expression of additional M1-associated genes (other than *Il12a* and *Tnf*) we analyzed gene expression of *Nos2* and *Il6* and observed their synergistic expression with CEP and Pam3CSK4 co-stimulation (Fig. 5E and 5F). We tested several other M1 genes (e.g., *Il12b, Cxcl1, Cxcl3*) and found similar results (data not shown). These results suggest that CEP has a unique ability to augment M1 polarization induced by Pam3CSK4.

### CEP potentiates Pam3CSK4-inflammatory signaling, but not other M1-inducing agonists

**Figure 6:**
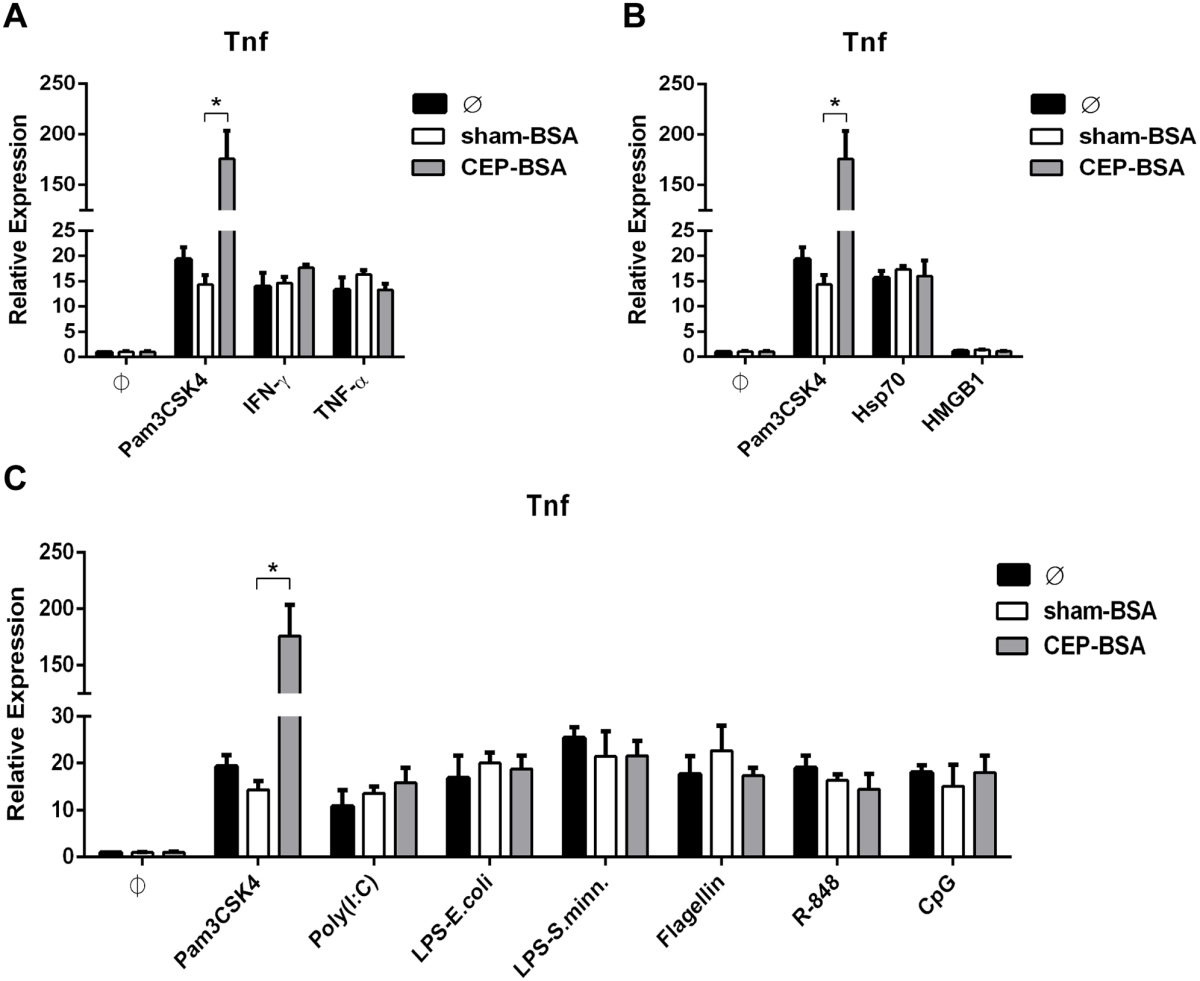
CEP synergizes specifically with TLR2 agonists and not inflammatory cytokines, DAMPs, or other TLR-ligands. (**A, B, C**) BMDMs were co-stimulated as indicated for 9 hours as indicated and *Tnf* mRNA was assessed by qPCR. (**A**) IFN-γ (1.5 ng/mL) or TNF-α (2.0 ng/mL) was combined with either sham-BSA or CEP-BSA (10 µg/mL) (n = 3) (**B**) Hsp70 (5 µg/mL) or HMGB1 (30 µg/mL) was combined with either sham-BSA or CEP-BSA (10 µg/mL) (n = 3) (**C**) Poly(I:C) (0.8 µg/mL), LPS-E.Coli (0.75 ng/mL), LPS-S.minn (0.5 ng/mL), Flagellin (12 ng/mL), R-848 (1.7 ng/mL), or CpG (60 nM) was combined with either sham-BSA or CEP-BSA (10 µg/mL) (n = 5). As a reference, the same stimulation experiment with Pam3CSK4 (1.0 ng/mL) (± sham-BSA or CEP-BSA [10 µg/mL]) is plotted in each figure (n = 5). *P<0.05. Results are mean +/− SEM.

The results thus far demonstrate CEP’s ability to enhance Pam3CSK4-induced M1 polarization of macrophages. However, it is possible that CEP might enhance any M1-inducing stimulus (not just Pam3CSK4). In order to explore this possibility, we co-stimulated BMDMs with CEP-BSA and various M1-activating agents. We tested several classes of M1-inducing agents: inflammatory cytokines, DAMPs, and TLR-ligands. For all these experiments, we previously performed titration experiments with each specific agonist to determine a sub-maximal concentration that induced gene expression of *Tnf* (and other genes) to a level that approximated the expression induced by 1.0 ng/mL of Pam3CSK4. An example titration of LPS is demonstrated in Figure S3. Co-stimulation experiments between CEP and the various M1-activating agents was performed for more than one concentration of the M1-activating agent, but only the concentration that mimics 1.0 ng/mL of Pam3CSK4 is depicted (Figure S4 demonstrates an example with LPS at different concentrations). Also, for simplicity only *Tnf* mRNA expression is depicted, but the gene-induction pattern was similar for *Il12a, Nos2,* and *Il6*. We first examined cooperation between CEP and inflammatory cytokines because this mimics the inflammatory microenvironment of certain disease states that are characterized by chronic low-grade inflammation and the absence of overt pathogens (e.g., atherosclerosis, AMD, cancer). As mentioned earlier, these microenvironments can also be associated with elevated CEP levels [10]. We observed that the *Tnf* gene expression elicited by sub-maximal concentrations of IFN-γ and TNF-α was not potentiated by co-stimulation with CEP-BSA (Fig. 6A). Next, we studied the relationship between CEP and DAMPs. DAMPs are important because, like inflammatory cytokines, they are present in diseased and damaged tissue. We examined two well known DAMPs, Hsp70 and HMGB1. Neither DAMP was able to synergize with CEP, as determined by gene induction of *Tnf* (Fig. 6B). Lastly, we examined synergy between CEP and TLR-agonists other than Pam3CSK4 (which is a TLR2-agonist). Specifically, we surveyed agonists for TLR3 (poly (I:C)), TLR4 (LPS derived from *Salmonella minnesota* and *Escherichia coli*), TLR5 (flagellin), TLR7 (R-848), TLR8 (R-848), and TLR9 (CpG). The results are depicted in Figure 6C and demonstrate the remarkable specificity between CEP and Pam3CSK4. Specifically, we observed that CEP failed to enhance *Tnf* gene-expression induced by TLR-ligands other than Pam3CSK4.

Taken together, these results demonstrate that CEP uniquely synergizes with Pam3CSK4. The lack of synergy between CEP and the other TLR-agonists is especially important because like Pam3CSK4, all these stimuli (with the exception of TLR3-agonists) induce *Tnf* expression vi*a* MyD88-dependent signaling cascades. Thus, CEP is not simply amplifying a generalized MyD88-dependent signaling pathway.

### CEP specifically cooperates with TLR2/TLR1-signaling

**Figure 7:**
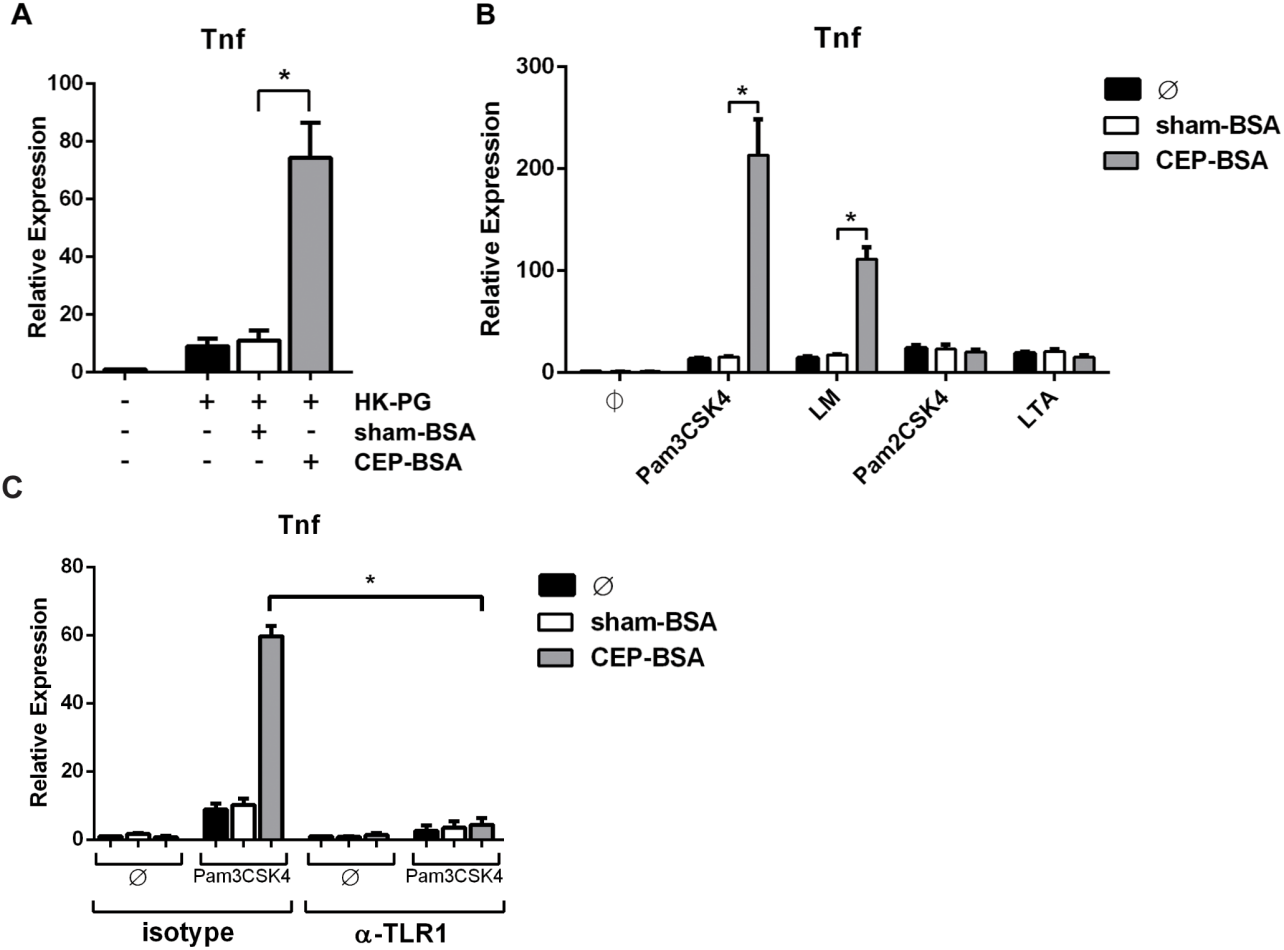
CEP cooperates specifically with the TLR2/TLR1 heterodimer. (**A, B**) BMDMs were co-stimulated for 9 hours as indicated and *Tnf* mRNA was assessed by qPCR. (**A**) Heat-killed (HK) *Porphyromonas gingivalis* (PG) (0.5 million/mL) were combined with either sham-BSA or CEP-BSA (10 µg/mL) (n = 3). (**B**) Pam3CSK4 (1.0 ng/mL), LM (30 ng/mL), Pam2CSK4 (0.15 ng/mL), or LTA (7 ng/mL) was combined with either sham-BSA or CEP-BSA (10 µg/mL) (n = 5). (**C**) THP-1 cells were pre-treated with 10 µg/mL anti-TLR1 antibody (or isotype) for 3 hours and then stimulated as indicated with sham-BSA (10 µg/mL), CEP-BSA (10 µg/mL), and/or Pam3CSK4 (1.0 ng/mL) for 9 hours followed by qPCR expression analysis for *Tnf* (n = 3). *P<0.05. Results are mean +/− SEM.

The results from the previous set of experiments point to a prominent role of TLR2 in CEP’s ability to influence inflammatory signaling. Thus we sought to determine if CEP can augment inflammatory signaling from different TLR2-agonists besides Pam3CSK4. We first examined heat-killed (HK) bacteria that are known to induce TLR2-signaling, specifically HK-*Porphyromonas gingivalis* (HK-PG) [25]–[28]. We observed synergistic gene induction of *Tnf* and other M1-associated genes in BMDMs co-stimulated with CEP-BSA and HK-PG (Fig. 7A and data not shown). This result demonstrates that CEP is able to potentiate inflammatory signaling from whole TLR2-activating bacteria and may have important implications in the context of low-grade bacteremia.

We next explored synergy between CEP and purified TLR2-agonists other than Pam3CSK4. TLR2, unlike the other TLRs, functions as a heterodimer forming either a TLR2/TRL1 or TLR2/TLR6 complex, allowing TLR2 to discriminate between different types of ligands [29], [30]. TLR2/TLR1 heterodimers bind triacylated lipoproteins (e.g., Pam3CSK4 and lipomannan [LM]) while TLR2/TLR6 heterodimers bind diacylated lipoproteins (Pam2CSK4, lipoteichoic acid [LTA]) [31]–[33]. We tested if CEP potentiated signaling from either TLR2/TLR1, TLR2/TLR6, or both by co-stimulating BMDMs with CEP-BSA and either Pam3CSK4, LM, Pam2CSK4, or LTA. After analyzing gene expression of *Tnf* and other M1-associated genes, we discovered that CEP only potentiated TLR2/TLR1-mediated signaling. These data clearly demonstrates that CEP enhanced the Pam3CSK4- and LM-induced gene expression of *Tnf* (Fig. 7B). In contrast, CEP offered no enhancement toward either Pam2CSK4- or LTA-induced gene expression (Fig. 7B). These results demonstrate a remarkable specificity of CEP toward enhancing TLR2/TLR1-signaling (and not TLR2/TLR6-signaling). In order to confirm the role of TLR1 in mediating the observed synergistic signaling we utilized a neutralizing TLR1 antibody. Given that this antibody is specific to human TLR1, we made use of the human macrophage cell line THP-1 for this experiment. As seen in Figure 7C, anti-TLR1 neutralizing antibody strongly attenuated CEP/Pam3CK4 synergistic signaling, confirming the requirement of TLR1. This result also importantly demonstrates that CEP’s ability to enhance TLR2/TLR1 signaling occurs in human macrophages as well.

## Discussion

Given the association of M1-macrophage accumulation and CEP-adduct formation in the retinas of AMD patients and CEP-immunized mice, we were motivated to understand the impact of CEP-adducts on macrophage innate signaling [11], [13], [16], [18]. In this report, we enhanced our mechanistic knowledge of CEP signaling by discovering an entirely novel and potent synergy between CEP and TLR2/TLR1 agonists. For instance, CEP enhanced Pam3CSK4-induced TNF-α protein secretion by 270-fold. Additionally, we observed a unique and specific partnership between CEP and TLR2/TLR1 agonists. Notably, Pam3CSK4 was unable to synergize with a different oxidized-lipid adduct (MDA-BSA). Moreover, CEP failed to potentiate signaling from an M2-stimulus and a variety of different M1-inducing agonists, including a variety of TLR-ligands (except select TLR2-agonists). Lastly, we found that CEP discriminates between TLR2 heterodimer complexes, selectively enhancing signaling from TLR2/TLR1.

Precisely how CEP augments TLR2/TLR1 signaling remains to be determined. We tested the possibility that CEP upregulates the surface expression of TLR2 and/or TLR1, but did not find this to be the case (data not shown). One key question is whether CEP itself functions as a TLR2 ligand as reported by Doyle et al. and West et al. [7], [15]. In such a scenario, CEP and Pam3CSK4 might cooperatively assemble upon the TLR2/TLR1 receptor complex to induce synergistic signaling, CEP could selectively recruit its partner to the TLR2/TLR1 heterodimer, or CEP might stabilize the Pam3CSK4-TLR2/TLR1 interaction. Alternatively, it is possible that CEP binds a receptor distinct from TLR2/TLR1, propagating a signaling cascade that converges intracellularly with TLR2/TLR1-signaling. Evidence for a CEP receptor other than TLR2 stems from the observation that CEP-induced platelet aggregation operates via TLR9 (and not TLR2) [14]. In the latter scenario, CEP-specific signaling may activate transcription factor networks (such as the AP-1 family) that cooperate with the TLR2/TLR1-induced pro-inflammatory gene expression (most likely mediated by NF-κB). Regardless of specifics, sustained presence of CEP in the microenvironment seems to be required for synergy, as washing out CEP abrogates its effect (Fig. S2). This could be explained by CEP internalization and quick intracellular degradation, or it may reflect its potential role in Pam3CSK4-TLR2/TLR1 complex assembly.

A key finding of this study is the discovery of synergism between a particular DAMP (i.e., CEP-adduct) and a particular PAMP (i.e., Pam3CSK4, LM). Although numerous studies document the synergy and/or tolerance induced by PAMP/PAMP-stimulations, few examples exist that demonstrate DAMP/PAMP synergy in macrophage cytokine production [34]–[36]. One such example is mmLDL, which was shown to synergize with low-doses of LPS [6]. Another example is the DAMP HMGB1, which synergizes with a variety of M1-inducing agonists including CpG (TLR9-agonist), cytokines (IL-1β, but not TNF-α), and Pam3CSK4 [37]. Another example is hemoglobin and its degradation product heme. Neither hemoglobin nor heme induce cytokine production by themselves (like CEP), but hemoglobin synergizes with TLR2-, TLR3-, TLR4-, TLR7-, and TLR9-agonists, while heme synergizes with TLR3-, TLR4-, and TLR7-agonists [38]. Thus, these DAMPs, unlike CEP, display some degree of promiscuity in synergizing with different classes of PAMPs.

In contrast to the findings of Doyle et al., we found that CEP-alone cannot prime the inflammasome for ATP-dependent IL-1β release. One possible explanation is that the albumin used by Doyle et al. contained trace TLR2-activating contaminants. Evidence for trace contaminants in their albumin is supported by their report, which documented a background level of IL-1β stimulation by the albumin alone. In fact, albumin alone induced IL-1β in a dose dependent manner [7]. It is possible this background level of stimulation represents low levels of TLR2-agonists that CEP synergizes with. As documented in this current report, we find that CEP combined with low-dose Pam3CSK4 does promote IL-1β release. Of note, our own previous studies that used albumin with background activity also showed that CEP “alone” induced inflammatory cytokines [22]. However, in light of the present work, we propose that CEP by itself does not induce inflammatory cytokine production, but rather functions to potentiate low-grade TLR2/TLR1 inflammatory signaling. This CEP–TLR2/TLR1 partnership may have important implications in aging and disease.

The presence of macrophages and elevated CEP levels are known to co-exist in the context of AMD and atherosclerosis. These pathological states are characterized by chronic low-grade inflammation, possibly mimicked by low-dose Pam3CSK4 stimulation. AMD and atherosclerosis have been linked with microbial exposure. For instance, *Porphyromonas gingivalis* (associated with periodontitis) and *Chlamydophila pneumoniae* exposure have been linked to atherosclerotic progression [39]–[41]. Interestingly, these two organisms signal via TLR2 [26], [42]–[44]. Additionally, atherosclerotic plaques have been found to contain TLR2-ligands and are abundant in TLR2-expression [45]–[47]. In fact, Pam3CK4 aids in atherogenesis in an animal model of atherosclerosis and we can speculate that CEP deposits in plaques may augment pathology [48]. AMD has been linked with periodontitis and *Chlamydophila pneumoniae* exposure [49]–[53]. An animal model of AMD demonstrates that *Chlamydophila pneumoniae* enhanced retinal damage, which was TLR2-dependent [54]. Aside from these specific pathogens, it is known that even healthy individuals have background levels of circulating microbes and routine tooth-brushing can introduce transient low-grade bacteremia [55]–[58].

In human disease, the potential for CEP/TLR2 synergy may reflect the balance between an individual’s particular microbiota, bacterial load, and CEP-abundance. Aging-related oxidative stress can induce tissue damage/inflammation and lipid peroxidation, promoting macrophage recruitment and CEP-formation. CEP by itself poses little risk of inducing inflammatory cytokines. However, if there is a chance encounter with a small amount of TLR2/TLR1-activating pathogens (or pathogen fragments), the macrophages can undergo a powerful synergistic activation that leads to the induction or exacerbation of pathological inflammation. Targeting CEP/TLR2 signaling with TLR2-antagonists may hold therapeutic potential. TLR2/TLR1 specific small molecule inhibitors have been recently identified and will be worth screening in animal models of AMD, angiogenesis, and atherosclerosis [59].

## Supporting Information

Figure S1 **Wild-type BALB/c and wild-type C57BL/6 BMDMs respond similarly to CEP-BSA and Pam3CSK4 co-stimulation. (A, B)** BMDMs from either wild-type BALB/c or wild-type C57BL/6 mice were stimulated as indicated with Pam3CSK4 (1.0 ng/mL), sham-BSA (10 µg/mL), and/or CEP-BSA (10 µg/mL) for 9 hours. *Tnf* or *Il12a* expression was assessed by qPCR (n = 3). *P<0.05. Results are mean +/− SEM.
(TIF)

Figure S2 **CEP-specific signaling is an active process and requires sustained CEP presence in the microenvironment.** BMDMs from BALB/c mice were stimulated as indicated with Pam3CSK4 (1.0 ng/mL), sham-BSA (10 µg/mL), and/or CEP-BSA (10 µg/mL) for up to 9 hours. Co-stimulation conditions in this experiment are similar to those used throughout this manuscript (9 hrs), whereas sequential stimulation was performed by pre-treatment with the indicated single agent for 3 hrs, followed by addition of the secondary agent for an additional 6 hrs. For the indicated samples (w.o.), the pre-treatment was removed (washed out) after centrifugation; fresh culture media was added to the wells, followed by addition of the secondary agent. Pam3CSK4 stimulation for 6 hrs serves as a temporal control. *Tnf* expression was assessed by qPCR (n = 3). *P<0.05. Results are mean +/− SEM.
(TIF)

Figure S3 **Stimulation with 0.75 ng/mL of LPS-E. Coli approximates stimulation with 1.0 ng/mL of Pam3CSK4.** BMDMs were stimulated with Pam3CSK4 or LPS-*E.Coli* at the indicated concentration for 9 hours. *Tnf* expression was assessed by qPCR (n = 3). Results are mean +/− SEM.
(TIF)

Figure S4 **CEP fails to potentiate various concentrations of LPS.** BMDMs were stimulated as indicated with Pam3CSK4 (1.0 ng/mL), LPS-*E.Coli* (at the indicated concentration), sham-BSA (10 µg/mL), and/or CEP-BSA (10 µg/mL) for for 9 hours. *Tnf* expression was assessed by qPCR (n = 3). *P<0.05. Results are mean +/− SEM.
(TIF)
