# Supplementary figures and images for: The Oxidative Stress Product Carboxyethylpyrrole Potentiates TLR2/TLR1 Inflammatory Signaling in Macrophages
Source: PLoS One. 2014 Sep 3;9(9):e106421. doi: 10.1371/journal.pone.0106421 (PMC4153630; doi:10.1371/journal.pone.0106421)

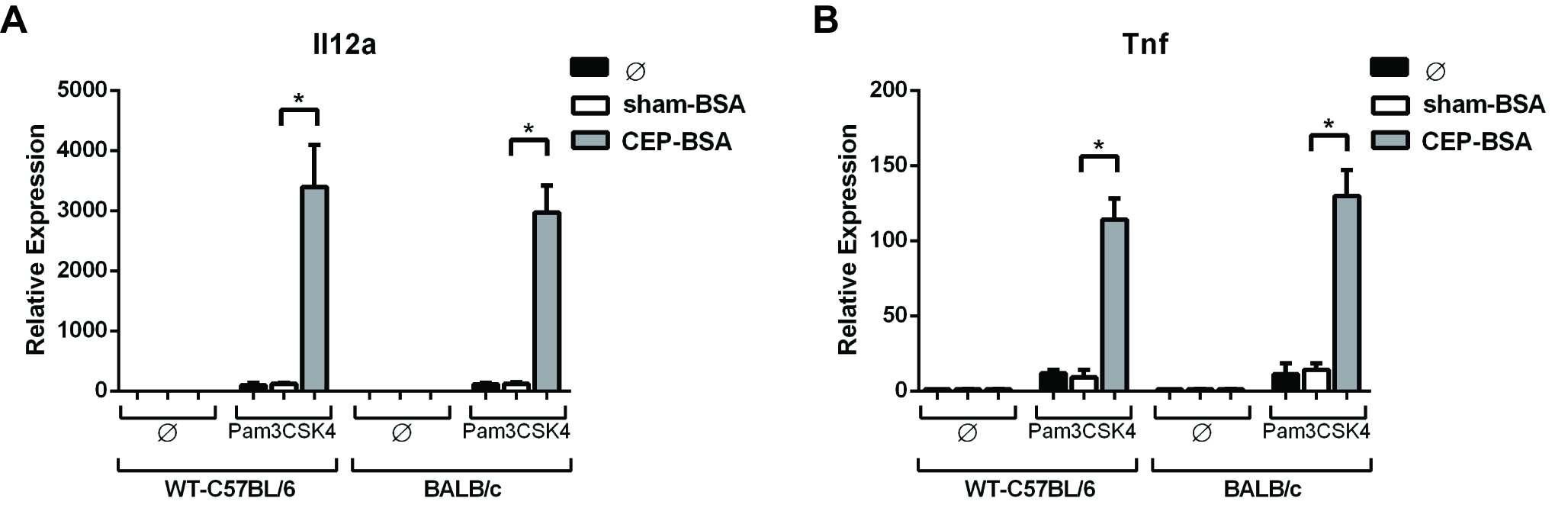

Supplement: Figure S1 — Wild-type BALB/c and wild-type C57BL/6 BMDMs respond similarly to CEP-BSA and Pam3CSK4 co-stimulation. (A, B) BMDMs from either wild-type BALB/c or wild-type C57BL/6 mice were stimulated as indicated with Pam3CSK4 (1.0 ng/mL), sham-BSA (10 µg/mL), and/or CEP-BSA (10 µg/mL) for 9 hours. Tnf or Il12a expression was assessed by qPCR (n = 3). *P<0.05. Results are mean +/− SEM. (TIF) [file pone.0106421.s001.tif]

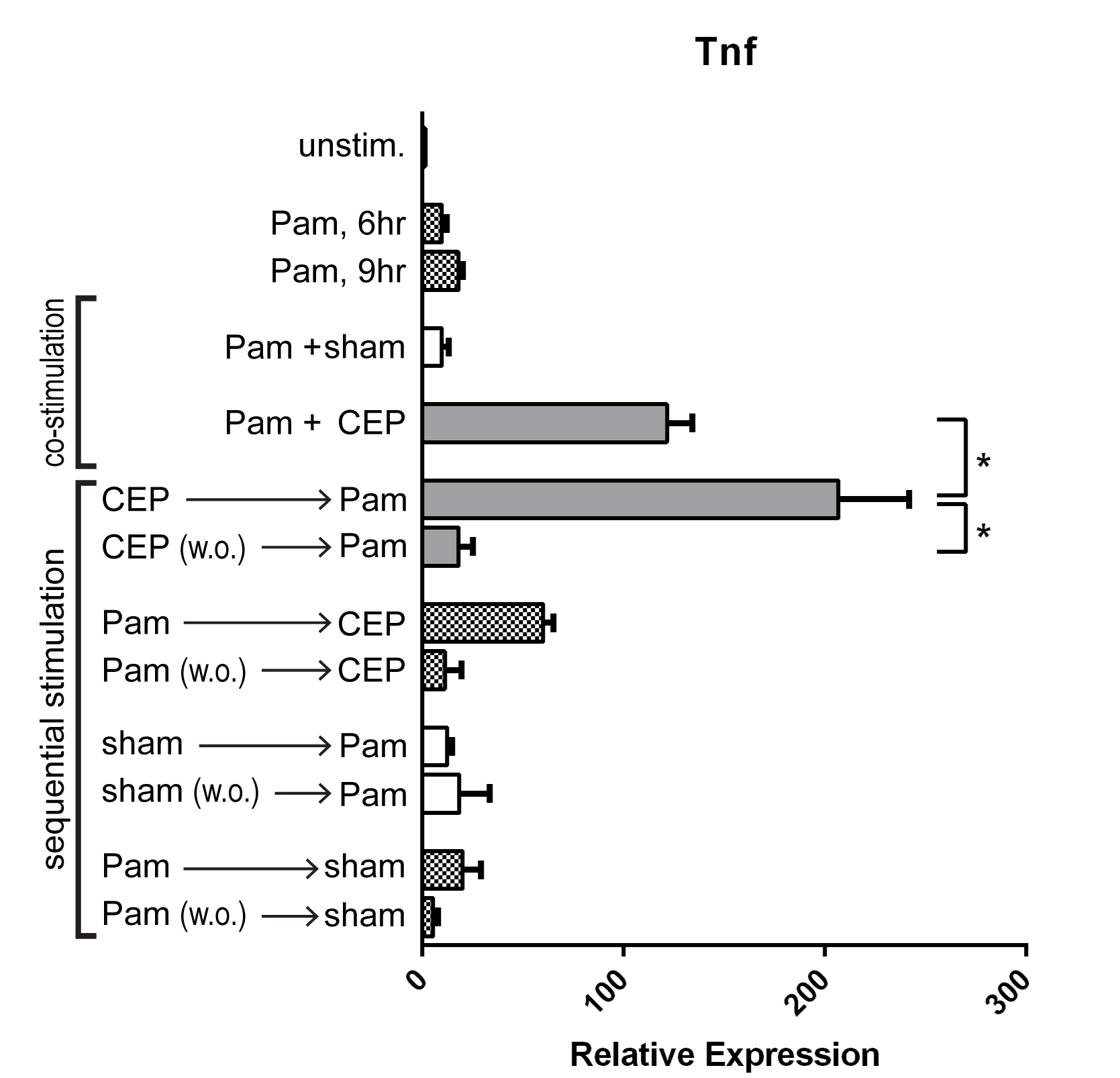

Supplement: Figure S2 — CEP-specific signaling is an active process and requires sustained CEP presence in the microenvironment. BMDMs from BALB/c mice were stimulated as indicated with Pam3CSK4 (1.0 ng/mL), sham-BSA (10 µg/mL), and/or CEP-BSA (10 µg/mL) for up to 9 hours. Co-stimulation conditions in this experiment are similar to those used throughout this manuscript (9 hrs), whereas sequential stimulation was performed by pre-treatment with the indicated single agent for 3 hrs, followed by addition of the secondary agent for an additional 6 hrs. For the indicated samples (w.o.), the pre-treatment was removed (washed out) after centrifugation; fresh culture media was added to the wells, followed by addition of the secondary agent. Pam3CSK4 stimulation for 6 hrs serves as a temporal control. Tnf expression was assessed by qPCR (n = 3). *P<0.05. Results are mean +/− SEM. (TIF) [file pone.0106421.s002.tif]

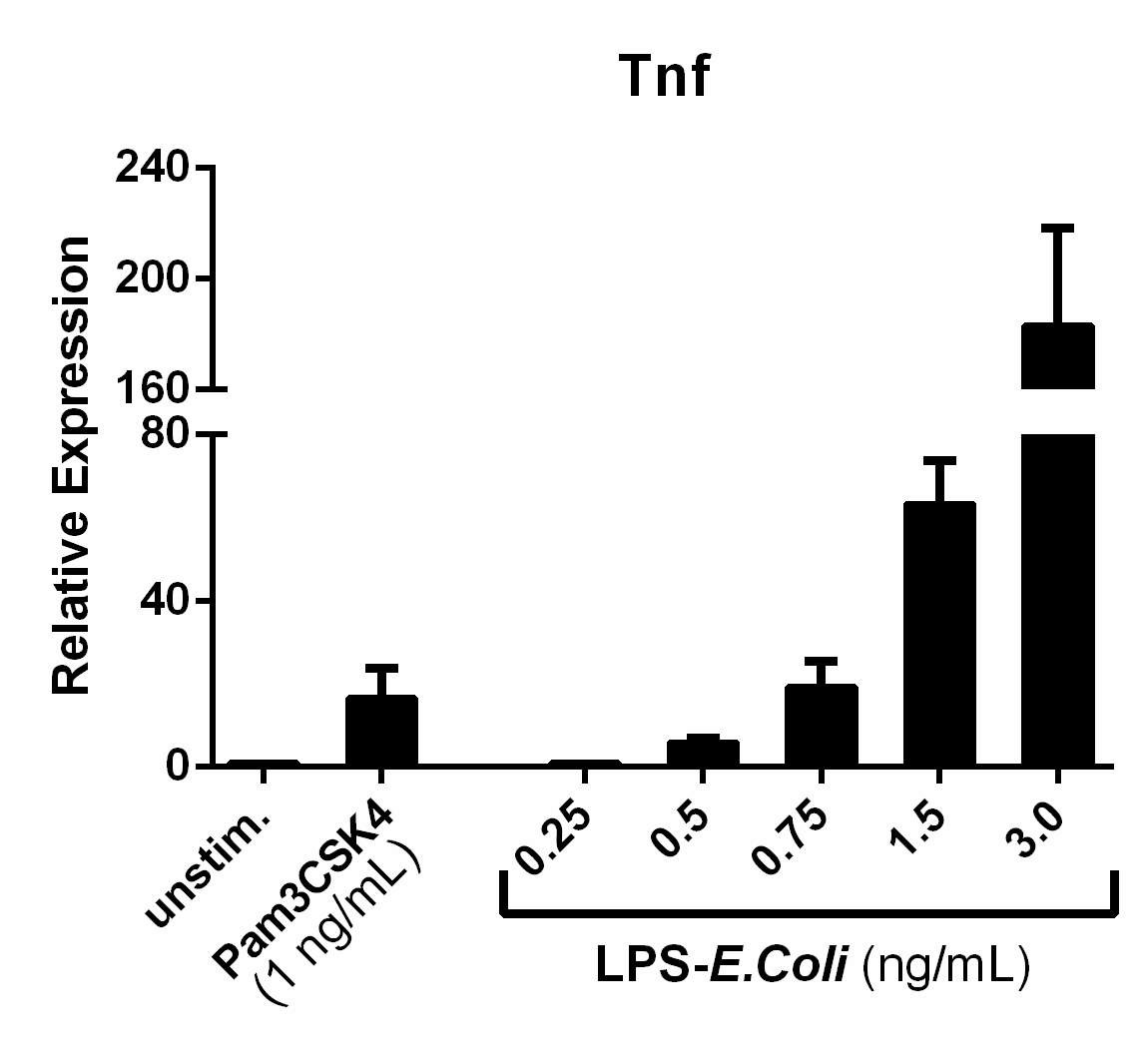

Supplement: Figure S3 — Stimulation with 0.75 ng/mL of LPS-E. Coli approximates stimulation with 1.0 ng/mL of Pam3CSK4. BMDMs were stimulated with Pam3CSK4 or LPS-E.Coli at the indicated concentration for 9 hours. Tnf expression was assessed by qPCR (n = 3). Results are mean +/− SEM. (TIF) [file pone.0106421.s003.tif]
